# Genome Segregation by the Venus Flytrap Mechanism: Probing the Interaction Between the ParF ATPase and the ParG Centromere Binding Protein

**DOI:** 10.3389/fmolb.2020.00108

**Published:** 2020-06-16

**Authors:** Marisa Caccamo, Aneta Dobruk-Serkowska, Fernando Rodríguez-Castañeda, Cecilia Pennica, Daniela Barillà, Finbarr Hayes

**Affiliations:** ^1^Faculty of Biology, Medicine and Health, The University of Manchester, Manchester, United Kingdom; ^2^Department of Biology, University of York, York, United Kingdom

**Keywords:** multidrug resistance, plasmid partition, ParF, ParG, ParA, *Escherichia coli*, segregation

## Abstract

The molecular events that underpin genome segregation during bacterial cytokinesis have not been fully described. The tripartite segrosome complex that is encoded by the multiresistance plasmid TP228 in *Escherichia coli* is a tractable model to decipher the steps that mediate accurate genome partitioning in bacteria. In this case, a “Venus flytrap” mechanism mediates plasmid segregation. The ParG sequence-specific DNA binding protein coats the *parH* centromere. ParF, a ParA-type ATPase protein, assembles in a three-dimensional meshwork that penetrates the nucleoid volume where it recognizes and transports ParG-*parH* complexes and attached plasmids to the nucleoid poles. Plasmids are deposited at the nucleoid poles following the partial dissolution of the ParF network through a combination of localized ATP hydrolysis within the meshwork and ParG-mediated oligomer disassembly. The current study demonstrates that the conformation of the nucleotide binding pocket in ParF is tuned exquisitely: a single amino acid change that perturbs the molecular arrangement of the bound nucleotide moderates ATP hydrolysis. Moreover, this alteration also affects critical interactions of ParF with the partner protein ParG. As a result, plasmid segregation is inhibited. The data reinforce that the dynamics of nucleotide binding and hydrolysis by ParA-type proteins are key to accurate genome segregation in bacteria.

## Introduction

Accurate genome segregation is an essential cellular process that guarantees the stable transmission of genetic material during cytokinesis (Bloom and Joglekar, 2010; Hirano, 2015). Despite the importance of bacteria in maintenance of the biosphere, in nutrient recycling, and in human and animal health, and disease, knowledge of the mechanisms that underpin genome maintenance in bacteria is very fragmented (Badrinarayanan et al., 2015). Nevertheless, low copy number plasmids that encode dedicated partition complexes have proven to be highly tractable and informative elements to probe the events leading to accurate bacterial genome segregation (Hayes and Barillà, 2006, 2010; Gerdes et al., 2010; Sengupta and Austin, 2011; Baxter and Funnell, 2014; Oliva, 2016).

The plasmid segregation complex (segrosome) typically comprises three components: a centromere-like site that is composed of repeat sequences whose numbers, lengths, and sequences differ between plasmids; a centromere binding protein that is specific for the cognate centromere; and, a motor protein that interacts with the DNA binding factor and which drives plasmid movement. The most widely-distributed motor proteins that mediate plasmid segregation are those of the ParA superfamily of Walker-type ATP-binding proteins that also are encoded widely by bacterial chromosomes. Despite their prevalence, the mechanism by which ParA proteins facilitate DNA segregation remains unclear. Numerous ParA homologs from diverse sources form higher-order structures upon ATP binding. Oligomerization is modulated by the cognate centromere binding protein and/or by DNA (Barillà et al., 2005, 2007; Leonard et al., 2005; Lim et al., 2005; Ebersbach et al., 2006; Bouet et al., 2007; Hatano et al., 2007; Machón et al., 2007; Pratto et al., 2008; Batt et al., 2009; Ringgaard et al., 2009; Ditkowski et al., 2010, 2013; Hui et al., 2010; Banigan et al., 2011; Dobruk-Serkowska et al., 2012; Kalliomaa-Sanford et al., 2012). A cycle of ParA assembly and disassembly within the nucleoid may promote the directed movement of plasmid DNA prior to cell division (Barillà et al., 2005; Ringgaard et al., 2009; McLeod et al., 2017). Another model suggests that the nucleoid provides a substructure for plasmid segregation by a diffusion-ratchet mechanism in which a gradient of ParA protein across the surface of the nucleoid promotes plasmid movement (Hatano and Niki, 2010; Vecchiarelli et al., 2010, 2014; Hwang et al., 2013). A variation of this model proposes that plasmids are segregated by recruitment to high-density regions within the nucleoid via interactions mediated by the ParA protein (Le Gall et al., 2016). Alternatively, a DNA-relay mechanism may utilize the elastic dynamics of the chromosome to convey the segregation complex between DNA regions via a ParA gradient (Lim et al., 2014).

The multidrug resistance plasmid TP228 replicates stably at low copy number in *Escherichia coli*. The TP228 segrosome comprises the *parH* centromere site; the ParG centromere binding protein; and the ParA homolog, ParF (Hayes, 2000; Barillà and Hayes, 2003). The *parH* site consists of an array of degenerate tetramer boxes interspersed by AT-rich spacers (Wu et al., 2011). The site is coated by ParG dimers each of which includes a C-terminal ribbon-helix-helix domain that is formed by the intertwining of a pair of monomers into a 2-fold symmetrical structure (Golovanov et al., 2003; Saeed et al., 2015). The ribbon-helix-helix domain mediates sequence-specific DNA binding (Carmelo et al., 2005; Zampini et al., 2009; Wu et al., 2011; Zampini and Hayes, 2012). This domain is linked to flexible N-terminal extensions that modulate DNA binding by ParG (Wu et al., 2011). The flexible tails in ParG also promote the formation of higher-order structures by ParF, and in addition harbor arginine finger-like motifs that enhance ATP hydrolysis by ParF (Carmelo et al., 2005; Barillà et al., 2007).

**Figure 1:**
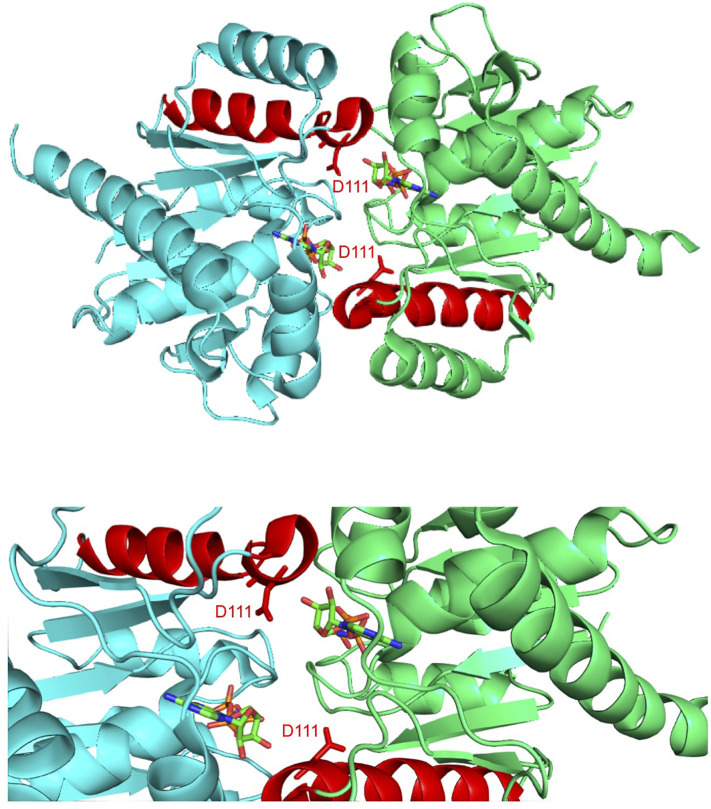
Co-crystal structure of the ParF dimer with AMPPCP (Schumacher et al., 2012). α-5 is highlighted in red with residue Asp-111, which is located at the C-terminal end of this helix, and AMPPCP shown as sticks. The *top* image shows the overall ParF dimer structure and the *bottom* image shows a zoom of the region that contains Asp-111. The images were made using PyMol (Delano, 2001).

The structures of ParF in complex with ADP and the non-hydrolyzable ATP analog AMPPCP in both cases comprise a single domain that consists of a central seven-stranded twisted β-sheet surrounded on each side by four α-helices (Schumacher et al., 2012). The protein is monomeric within the ParF-ADP complex which correlates with observations that ADP inhibits the production of higher-order structures by ParF (Barillà et al., 2005; Dobruk-Serkowska et al., 2012). In contrast with the ADP bound form of the protein, the ParF-AMPPCP structure is dimeric with the nucleotide molecules sandwiched between monomer subunits (Figure 1). Significantly, these subunits pack into dimer-of-dimer assemblies that in turn are organized into higher-order structures (Schumacher et al., 2012). Importantly, mutations at the interfaces of the ParF dimer-of-dimer concomitantly disrupted oligomerisation and DNA segregation which affirmed that plasmid partitioning by ParF necessitates the formation of higher-order structures by the protein (Schumacher et al., 2012).

Cycles of ATP binding and hydrolysis promote the oscillation of ParF between the poles of the nucleoid. The tethered ParG-*parH* complex is co-transported with ParF as it relocates. The tempo of this oscillatory pattern is key to effective segregation (McLeod et al., 2017). Moreover, in contrast with the diffusion-ratchet segregation model in which a gradient of ParA protein over the surface of the nucleoid has been suggested to promote plasmid transport, the dynamic ParF matrix instead invades the interior of the nucleoid. This three-dimensional meshwork acts as a “Venus flytrap” that captures and transports ParG-*parH* complexes, and therefore the attached plasmids, to the nucleoid poles. The plasmids are deposited at the poles as a result of localized dissolution of the ParF meshwork via ParG-promoted disassembly (McLeod et al., 2017). Here, we probe the role in plasmid segregation of a critical secondary structure element in the ParF protein. The data reveal that a single amino acid change that perturbs the molecular arrangement of the bound nucleotide moderates ATP hydrolysis by ParF, as well as the interaction of ParG *in trans* with the nucleotide binding pocket.

## Materials and Methods

### Bacterial Strains and Growth Conditions

*E. coli* DH5α (F^−^ *endA1 hsdR17* (${\text{r}}_{\text{K}}^{-}$ ${\text{m}}_{\text{K}}^{+}$) *supE44 thi-1 recA1 gyrA96* (Nal^r^) *relA1 deoR* Φ80*lacZ*ΔM15 Δ(*lacZYA-argF*) U169) (Hanahan, 1985) was used for plasmid propagation and gene cloning. High level gene expression and protein purification was performed with *E. coli* BL21 (DE3) (F^−^ *omp hsdSB* (${\text{r}}_{\text{B}}^{-}$${\text{m}}_{\text{B}}^{-}$) *gal dcm*) (Studier and Moffatt, 1986). Strain BR825 is a *polA* mutant (Ludtke et al., 1989) that was used in plasmid segregation assays. *E. coli* SP850 (λ^−^ *e14*^−^ *spoT1* Δ(*cya1400::kan*) *thi-1*) carries a 200 bp deletion within the *cya* gene (Shah and Peterkofsky, 1991) and was used in two-hybrid assays. Strains were propagated in Luria-Bertani (LB) broth or agar medium at 37°C unless otherwise stated. Appropriate antibiotics for plasmid selection were included when required (ampicillin, 100 μg/ml; chloramphenicol, 10 μg/ml).

### Plasmids

Plasmid pFH547 consists of the *parFGH* cassette of multiresistance plasmid TP228 cloned in the plasmid partition vector pFH450. The latter is a derivative of pALA136 into which a multiple cloning site was inserted (Martin et al., 1987; Hayes, 2000). Site-specific mutations in *parF* were introduced into pFH547 by overlap extension PCR (Higuchi et al., 1988). Mutated genes were amplified from pFH547-based plasmids and cloned in pT18 and pT25 two-hybrid vectors (Karimova et al., 1998) and in the pET-22b(+) expression vector (Novagen) as outlined previously (Barillà and Hayes, 2003). All mutations were verified by sequencing. Plasmids comprising the *parG* gene cloned in two-hybrid vectors and in expression vectors were described elsewhere (Barillà and Hayes, 2003).

### Plasmid Partition Assays

Plasmid partition vector pFH450 includes the moderate copy number pMB1 replicon and the low copy number P1 replicon as well as a chloramphenicol resistance determinant. The pMB1 replicon allows facile propagation of the plasmid and its derivatives in strain DH5α, whereas the plasmid switches to the low copy number P1 replicon in the BR285 strain that lacks the *polA* gene product (DNA polymerase I) that is essential for replication by the pMB1 replicon. The FH450 plasmid is segregationally unstable in this background which is detectable as rapid plasmid loss in the absence of chloramphenicol selective pressure. The vector is stabilized by the insertion of the *parFGH* cassette (Hayes, 2000). Partition assays were performed as outlined previously (Martin et al., 1987; Hayes, 2000). In brief, pFH450-based plasmids were transformed into strain BR825 with selection on LB agar containing chloramphenicol. Eight colonies from this transformation were streaked on chloramphenicol plates to ensure plasmid establishment. Following ~16 h of growth, one colony from each of these eight streaks was inoculated on LB plates without antibiotic to allow for plasmid retention or loss during the ~20–25 generations required for subsequent colony formation. One colony from each of these streaks was again streaked on non-selective plates to separate cells that had retained or lost the plasmid during the first round of non-selective growth. Eight colonies from each of the eight streaks were tested for plasmid retention by replica plating onto LB agar with and without chloramphenicol. Growth was assessed following ~16 h incubation. Assays were performed at least in triplicate and are expressed as mean values with typical standard deviations of ~10%.

### Two-Hybrid Assays

Protein-protein interaction studies *in vivo* utilized the two-hybrid assay developed by Karimova et al. (1998) that has been used previously to monitor ParF self-association and the ParF-ParG interaction (Barillà and Hayes, 2003; Carmelo et al., 2005; Barillà et al., 2007; Dobruk-Serkowska et al., 2012). Test genes were fused in-frame with genes that specify the T18 and T25 fragments of *Bordetella pertussis* adenylate cyclase. When the encoded test proteins interact, the T18 and T25 fragments are brought in sufficiently close proximity to reconstitute adenylate cyclase activity in a *cya* mutant of *E. coli*. Adenylate cyclase function is monitored indirectly by assaying β-galactosidase activity (Karimova et al., 1998). Plasmid pairs producing fusions of the T18 or T25 subunits with either wild-type or mutated ParF or with ParG were cotransformed in strain SP850. Self-association of ParF or the interaction with ParG was assessed by β-galactosidase assays on cultures grown at 30°C for ~16 h. Results are averages of at least three independent tests with typical standard deviations <10%.

### Protein Purification and Biochemical Analysis

Wild-type and mutant ParF proteins and ParG protein were overproduced using pET-22b(+) expression plasmids and were purified as His-tagged derivatives as described previously (Barillà and Hayes, 2003). ATPase assays were conducted with [α-^35^S]ATP, analyzed by thin layer chromatography, and quantified as detailed elsewhere (Barillà et al., 2005). In brief, ParF in the absence or presence of ParG at the concentrations indicated in figure legends, was incubated with 1 μCi of [α-^35^S]ATP (1250 Ci/mmol) and unlabeled ATP at the concentrations indicated in figure legends in 30 mM Tris-HCl pH 7.5, 100 mM KCl, 5 mM MgCl_2_, 2 mM DTT in a final volume of 16 μl at 30°C for 60 min. Aliquots (2.5 μl) were applied to polyethyleneimine cellulose plates that had been prerun in water, dried and then subjected to TLC with 0.5 M KH_2_PO_4_ (pH 3.5) as buffer. The plates were dried and exposed to Kodak BioMax MR film.

Higher-order assembly of wild-type and mutant ParF proteins was assessed by sedimentation assays as described previously (Barillà et al., 2005). Briefly, ParF with or without ParG at the concentrations indicated in figure legends, was incubated in 30 mM Tris-HCl pH 8.0, 100 mM KCl, 2 mM DTT, 10% glycerol in a volume of 60 μl in the absence or presence of nucleotides (2 mM) and MgCl_2_ (5 mM) for 10 min at 30°C. Reactions were centrifuged for 30 min at 4°C at 20,800 × *g*. 20 μl of the supernatant was collected for SDS-PAGE analysis, 10 μl were retained for Bradford quantitation, and the remaining supernatant was carefully aspirated. The pellet was resuspended in 15 μl of water. The supernatant (20 μl; 33%) and pellet (15 μl; 100%) fractions were analyzed by SDS-PAGE and Coomassie Blue staining. Protein bands were quantitated with ImageJ (National Institutes of Health, Bethesda, MD, USA).

### Biophysical Measurements

The ATP binding activity of wild-type and mutated ParF proteins was determined by anisotropy measurements of the fluorescent ATP analog MANT-ATP as outlined previously (Dobruk-Serkowska et al., 2012). Briefly, fluorescence anisotropy measurements were made with a Jovin-Yvon Horiba Fluoromax-3 spectrofluorimeter in a quartz microcuvette in a total volume of 150 μl in a buffer comprising 20 mM HEPES, 150 mM NaCl, 1 mM MgCl_2_, pH 7.0. The excitation wavelength (λ_ex_) and emission wavelengths (λ_em_) were 356 and 442 nm, respectively. The ParF concentration was increased from 0.25 to ~5 μM whereas the MANT-ATP concentration was 0.9 μM. Ten measurements of fluorescence anisotropy were taken for each protein increment and the average value was plotted against ParF concentration. Due to potential hydrolysis of MANT-ATP by ParF and mutant proteins during measurements (Dobruk-Serkowska et al., 2012), binding assays also were done with the non-hydrolyzable MANT-ATPγS analog using the same conditions as for MANT-ATP.

Circular dichroism (CD) spectroscopy was performed with ParF solutions diluted to ~10 μM in 10 mM NaCl, 5 mM sodium phosphate pH 7.0. Ellipticity was determined in a quartz cell with a 0.5 cm path length (Fothergill et al., 2005). CD measurements were performed with a Jasco J-810 spectropolarimeter scanning from 190 to 260 nm at 25°C. Scans are the averages of at least three accumulations and were corrected against buffer-only spectra. Data were analyzed on the DichroWeb website (Lobley et al., 2002; Whitmore and Wallace, 2004) using CONTIN and CDSSTR software (Provencher et al., 1981; Compton and Johnson, 1986; Sreerama and Woody, 2000).

## Results

### Alanine Scanning Mutagenesis of Helix α5 in ParF

ParF co-structures with ADP and with the non-hydrolyzable AMPPCP nucleotides are available. The nucleotides in both co-structures are embedded within a surface-exposed cavity of ParF. This niche is formed by residues from the Walker A nucleotide binding motif and other amino acids which make tight contacts with the nucleotide and with a hexacoordinated magnesium ion (Schumacher et al., 2012). However, the ADP and AMPPCP ribose moieties adopt different puckers in the ParF structures: the ribose of the bound AMPPCP molecule assumes a C3′-*endo* conformation whereas a C2′-*endo* conformation is evident in the ParF-ADP structure. The C3′ conformation is not viable in the latter as this configuration would result in steric clash. The distance between adjacent phosphorus atoms and the orientation of these atoms relative to the sugar and bases differ significantly in these two ribose configurations. Thus, the distinctive nucleotide sugar puckers in the ADP- and AMPPCP-bound states may be a critical factor in determining whether the assembly of higher-order structures by ParF is antagonized by ADP or promoted by ATP (Schumacher et al., 2012).

Alpha-helix 5 (α5; residues 111–123) is of particular significance in the ParF structure as residue Asp-111, which is positioned at the N-terminal end of this element, makes key contacts with the ribose O3′ hydroxyl that stabilizes the C3′-*endo* conformation when ParF is bound to AMPPCP (Figure 1). Asp-111 is highly conserved among ParF homologs which attests further to the importance of this residue (Schumacher et al., 2012). Moreover, the C-terminal end of α5 forms a major part of one of two interfaces that underpin the ParF oligomer observed in complexes with AMPPCP. This interface is formed by interactions between residues 61–71 in α3 with 117–129 partly in α5, and 2-fold related contacts between residues 87–98 in α4 (Schumacher et al., 2012). In view of the importance of α5, and particularly of residue Asp-111, to the structure and function of ParF, the contribution of this element was probed further here.

**Figure 2:**
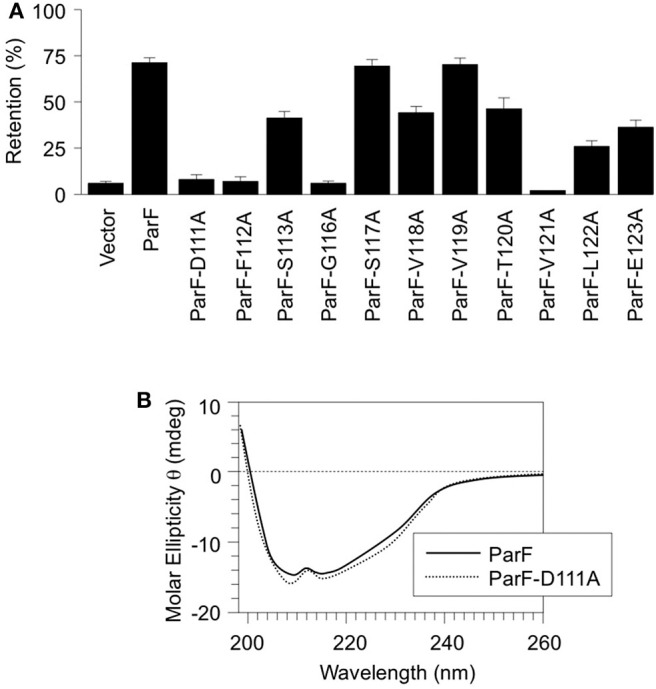
Alanine scanning mutagenesis of α5 in ParF. **(A)** Segregation assays using pFH547-based plasmids that contain the *parFGH* cassette cloned in the pFH450 partition assay vector. Plasmids encoded either wild-type ParF or ParF derivatives with the indicated site-specific mutations. Partition assays are means of at least three independent tests with typical standard deviations of <10%. **(B)** Far UV CD spectra of wild-type ParF (solid line) and ParF-D111A (dashed line). The differences in the spectra between the wild-type and mutated proteins are within the reproducibility limits of the technique. The CD spectrum of wild-type ParF was reported previously (Dobruk-Serkowska et al., 2012).

Amino acids 111–123 that comprise α5 were subjected to alanine scanning mutagenesis (Cunningham and Wells, 1989). Thus, the codon for each residue was changed independently by site-directed mutagenesis to an alanine codon, except at positions 114 and 115 both of which specify alanine in the wild-type protein. The effects of these alterations on ParF-mediated plasmid segregation were assessed (Figure 2A). The wild-type segregation module conferred ~70% plasmid retention in the assay whereas the vector without a partition cassette showed <5% retention. These values correspond to those described previously (Hayes, 2000; Fothergill et al., 2005; Barillà et al., 2007; Dobruk-Serkowska et al., 2012; Schumacher et al., 2012; Saeed et al., 2015). The S117A and V119A changes in α5 of ParF were innocuous and mutations at positions 113, 118, 120, 122, and 123 conferred partial segregation defects. In contrast, the D111A, F112A, G116A, and V121A mutations exerted potent effects and reduced plasmid maintenance levels to those of the empty vector (Figure 2A). These data support the functional importance of α5 in ParF-directed segregation. As the mutation of Asp-111 imparted a strong partitioning defect and as this residue makes vital interactions with the ribose moiety that stabilizes the C3′-*endo* conformation when ParF is in contact with the ATP analog AMPPCP (Figure 1), the ParF-D111A protein was purified and characterized further. CD analysis revealed that the gross structure of the protein was not disrupted appreciably by the D111A mutation (Figure 2B).

### The D111A Mutation Perturbs ATP Hydrolysis by ParF

**Figure 3:**
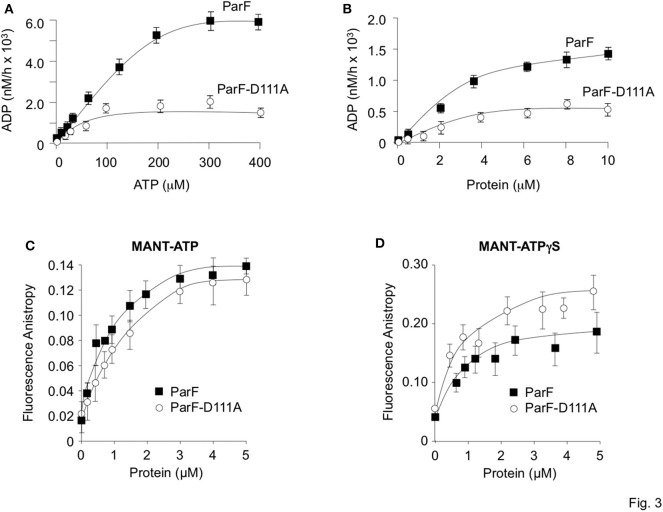
Comparative ATP hydrolysis and binding assays of wild-type ParF and ParF-D111A. **(A)** ATP hydrolysis is plotted with proteins (4 μM) at 0–400 μM ATP concentrations. **(B)** ATP hydrolysis is plotted with ATP (5 μM) at 0–10 μM ParF or ParF-D111A concentrations. **(C)** Anisotropy changes when MANT-ATP (0.9 μM) was titrated with increasing concentrations of wild-type ParF (filled squares) and ParF-D111A (open circles) proteins. **(D)** Anisotropy changes when MANT-ATPγS (0.9 μM) was titrated with increasing concentrations of wild-type ParF (filled squares) and ParF-D111A (open circles) proteins. The average fluorescence anisotropy values for 10 measurements for each point are shown in **(C,D)**.

ATP binding elicits the assembly of ParF into higher-order assemblies. The formation of these structures is regulated by the protein's weak ATPase activity (K_0.5_ ~100 μM) that generates an ADP-bound form of ParF which antagonizes assembly (Barillà et al., 2005). Mutations in ParF that either inhibit or enhance ATP binding or hydrolysis interfere both with the generation of higher-order ParF assemblies and with plasmid maintenance which confirm the crucial role of nucleotide binding in ParF meshwork formation and in segregation (Barillà et al., 2005; Dobruk-Serkowska et al., 2012). The ATPase kinetics of ParF-D111A were compared with those of the wild-type protein under *in vitro* conditions that were used previously to characterize ParF and other mutant derivatives. First, the proteins were tested at fixed concentrations (4 μM) with titrations of ATP up to 400 μM (Figure 3A). Wild-type ParF displayed the hyperbolic curve previously noted with maximal activity at ~250 μM ATP (Barillà et al., 2005; Dobruk-Serkowska et al., 2012). However, ATP hydrolysis was impaired in ParF-D111A: the protein showed a modest increase in activity at ATP concentrations up to ~100 μM but, unlike wild-type ParF, hydrolysis was not enhanced at higher nucleotide concentrations (Figure 3A). Second, a fixed ATP concentration (5 μM) was used in reactions with up to 10 μM ParF or ParF-D111A (Figure 3B). Hydrolysis was enhanced with increasing concentrations of the wild-type protein and tended toward a plateau at ~6 μM ParF. ParF-D111A displayed weaker ATPase activity than the wild-type protein using the same protein concentration range with maximal ADP production less than half that observed with wild-type ParF (Figure 3B).

The nucleotide binding dynamics of ParF and ParF-D111A were monitored by fluorescence anisotropy using the fluorescent ATP analog MANT-ATP (0.9 μM) titrated with the two proteins (Figure 3C). At low concentrations of the wild-type protein, MANT-ATP anisotropy elevated rapidly followed by a more gradual increase with an apparent *K*_*d*_ of ~0.5 μM which matches the value determined previously in anisotropy experiments with ParF (Dobruk-Serkowska et al., 2012; Schumacher et al., 2012). The ParF-D111A protein showed a very similar binding curve (Figure 3C). Fluorescence anisotropy studies also were performed with the non-hydrolyzable analog MANT-ATPγS (Figure 3D). As there essentially is no hydrolysis within the time scale of the experiment, MANT-ATPγS anisotropy traces represent the true ligand binding curves. Wild-type ParF and ParF-D111A each elicited a rapid initial increase in MANT-ATPγS anisotropy. Values with ParF leveled off at concentrations ~2 μM, yielding an apparent *K*_*d*_ of ~0.4 μM which is similar to that determined for MANT-ATP (Figure 3C) and to that established previously for ParF with MANT-ATPγS (Dobruk-Serkowska et al., 2012). Although the binding curve for ParF-D111A with MANT-ATPγS was slightly more shallow than the trace for ParF, the patterns were very similar with an apparent *K*_*d*_ (~0.5 μM) for ParF-D111A that was very close to that for the wild-type protein. Overall the fluorescence anisotropy experiments showed that both ParF and ParF-D111A bind MANT-ATP and non-hydrolyzable MANT-ATPγS similarly. Thus, the impaired ATPase activity of ParF-D111A is not due principally to a defect in nucleotide binding, but instead arises from a decreased rate of ATP hydrolysis which correlates with the role of Asp-111 in stabilizing the C3′-*endo* conformation when ParF is bound to AMPPCP (Schumacher et al., 2012).

### The D111A Mutation Poisons the Function of a ParF Hyperactive ATPase Mutant

**Figure 4:**
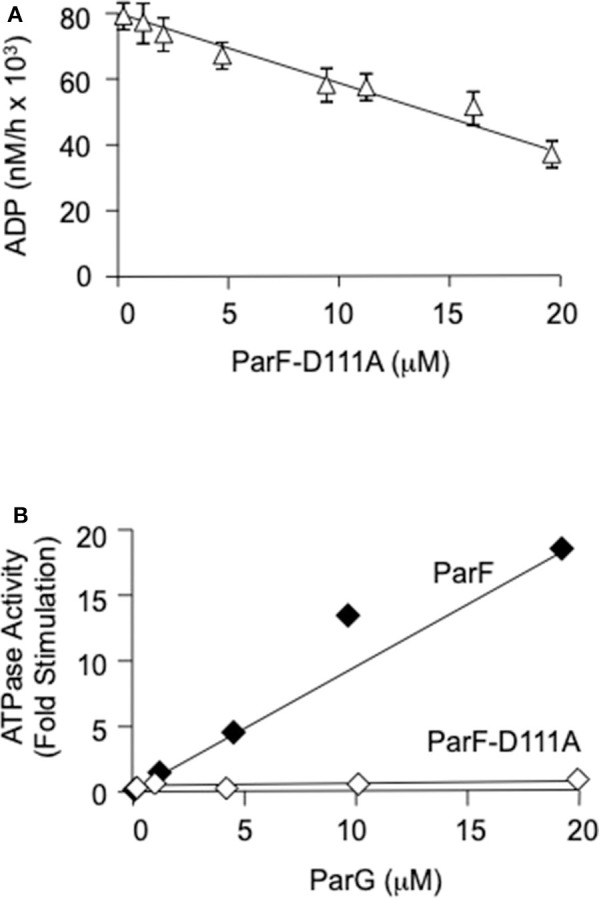
**(A)** Poisoning of the ATPase hyperactivity of ParF-P104A by D111A using 200 μM ATP. ParF-104A (4 μM) and ParF-D111A (0–20 μM) were added sequentially to the reaction buffer followed by the immediate addition of nucleotide. The results are averages of at least three replicates. **(B)** The ATPase activity of ParF-D111A is not stimulated by ParG. Levels of ATP hydrolysis driven by ParF ParF-D111A as a function of ParG concentration are shown. ParF proteins were used at 1 μM and ParG at 0-20 μM with 5 μM ATP. The data are expressed as fold stimulation of ATPase activity compared with basal activity without added ParG.

Residue Pro-104 is conserved in ParF and related members of the ParA superfamily, but is located distantly in the primary sequence from canonical nucleotide interaction motifs (Hayes, 2000). The residue is part of a proline-rich patch in ParF that inserts into a niche near to the ATP binding pocket of the neighboring subunit in ParF dimers (Schumacher et al., 2012). Although the role of this patch awaits elucidation, the P104A mutation induces ATPase hyperactivity in ParF that results from reorganization of the catalytic pocket. The mutation is thought to modify nucleotide access and stability in the pocket (Dobruk-Serkowska et al., 2012). Mixing experiments were conducted in which purified ParF-P104A (4 μM) was co-incubated with increasing concentrations of ParF-D111A (0–20 μM) in the presence of 200 μM ATP (Figure 4A). The ParF-P104A protein alone showed the characteristic ATPase hyperactivity described previously which manifested as an ~20-fold increase in ADP production compared to wild-type ParF (Dobruk-Serkowska et al., 2012). This activity was ablated modestly by ParF-D111A which reduced nucleotide hydrolysis by ParF-P104A by approximately half at the highest concentration of ParF-D111A that was tested. The data demonstrate that ParF-D111A interacts with ParF-P104A to impair the hyperactive ATPase properties of the latter, potentially through the formation of assorted dimers. Moreover, the results confirm that the D111A mutation does not grossly perturb the self-association properties of ParF.

### The D111A Mutation Impairs the Interaction of ParF With ParG

**Figure 5:**
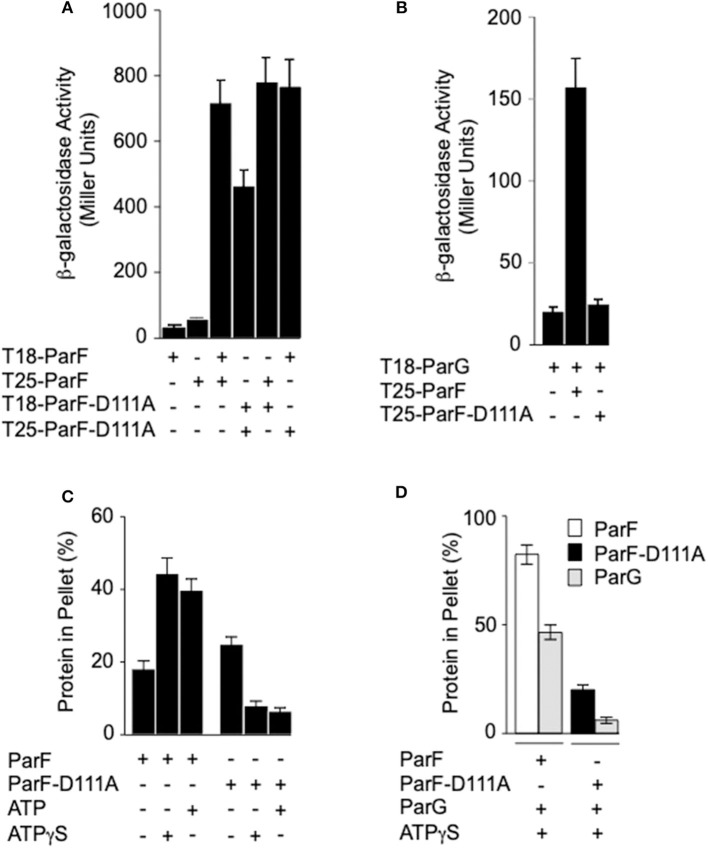
Effects of the D111A mutation in ParF on protein-protein interactions and ParF assembly into higher-order structures. **(A)** Two-hybrid analysis of ParF-D111A self-association and interaction with wild-type ParF. Fusions of wild-type or ParF-D111A to the T18 and T25 subunits of adenylate cyclase were tested in *E. coli* SP850. **(B)** Two-hybrid analysis of ParF-D111A interaction with ParG. Fusions of wild-type ParF or ParF-D111A to the T25 subunit of adenylate cyclase were tested with a fusion of ParG to the T18 subunit in *E. coli* SP850. Results in panels A and B are means of at least three independent tests with typical standard deviations <10%. **(C)** Kinetics of wild-type ParF and ParF-D111A assembly into higher-order species. Sedimentation assays were performed in which proteins (4–8 μM) were incubated in the absence (–) or presence of nucleotides (2 mM) for 10 min at 30°C, and the reactions were then centrifuged. In all, 100 and 33%, respectively, of the pellet and supernatant fractions were resolved on a 12% SDS gel and stained with Coomassie Blue (Barillà et al., 2005). The percentages of ParF and ParF-D111A proteins detected in the pellet fractions are shown. **(D)** Co-sedimentation assays of wild-type ParF and ParF-D111A with ParG. Sedimentation assays were performed in which ParF proteins (4-8 μM) and ParG (~5 μM) were incubated in the absence (–) or presence of ATPγS (2 mM) for 10 min at 30°C, and the reactions were then centrifuged. In all, 100 and 33%, respectively, of the pellet and supernatant fractions were resolved on a 12% SDS gel and stained with Coomassie Blue. The percentages of ParF, ParF-D111A, and ParG proteins detected in the pellet fractions are shown.

Self-association of ParF and the interaction between ParF and the partner segregation protein ParG can be monitored in two-hybrid assays *in vivo* (Barillà and Hayes, 2003; Fothergill et al., 2005; Barillà et al., 2007). The proteins under study are fused with the T18 and T25 polypeptide fragments that form the catalytic domain of the adenylate cyclase protein of *B. pertussis*. If the test proteins interact, the T18 and T25 fragments are brought into sufficiently close spatial proximity that adenylate cyclase enzymatic activity is reconstituted and therefore cyclic AMP synthesis is restored in an *E. coli cya* mutant (Karimova et al., 1998). Cyclic AMP triggers the expression of numerous catabolic operons, including the lactose operon. Therefore, the interaction between the test proteins can be assessed semi-quantitatively by measuring β-galactosidase levels (Karimova et al., 1998). The unfused adenylate cyclase polypeptides or ParF fused to one fragment, but not to the second domain, produced <55 β-galactosidase units. In contrast, self-association of wild-type ParF fused to the T18 and T25 polypeptides generated ~700 β-galactosidase units (Figure 5A) as noted previously (Dobruk-Serkowska et al., 2012). The D111A mutation decreased β-galactosidase levels by approximately one-third when the alteration was included in both the ParF-T18 and ParF-T25 fusions. However, heterodimerization of wild-type ParF with ParF-D111A when fused to T18 and T25, respectively, or vice versa, resulted in similar β-galactosidase values to that observed for homodimerization of the wild-type protein (Figure 5A).

The effect of the D111A mutation in ParF on the interaction with ParG also was examined in two-hybrid assays. In contrast to the modest effect that the mutation exerted on ParF self-association, the D111A change abolished detectable association of ParF with ParG *in vivo* (Figure 5B). Thus, the two-hybrid analyses reveal that ParF-D111A is specially impaired in interaction with the partner segregation protein ParG. Thus, in parallel with the perturbation in nucleotide binding described above, the D111A mutation may disrupt the nucleotide binding pocket in ParF sufficiently that the interaction with the arginine-like finger in ParG is compromised thereby leading to loss of detectable interaction with the latter in two-hybrid tests (Figure 5B). Accordingly, whereas the ATPase activity of wild-type ParF was stimulated up to ~20-fold by ParG as observed previously (Barillà et al., 2005, 2007; Dobruk-Serkowska et al., 2012), the ParF-D111A protein was unresponsive to ParG stimulation (Figure 4B).

### Self-Association Properties of ParF-D111A *in vitro*

Purified ParF self-associates modestly in the absence of added nucleotide *in vitro*. Self-association is enhanced strongly by ATP, but is inhibited by ADP, which indicates that the cycle of ParF assembly and disassembly is regulated in part by hydrolysis of bound nucleotide (Barillà et al., 2005, 2007; Machón et al., 2007; Dobruk-Serkowska et al., 2012; Schumacher et al., 2012). The formation of higher-order ParF complexes is monitored by sedimentation assays in which the protein is incubated with or without added nucleotide, and separated by centrifugation into pellet, and supernatant fractions that harbor oligomeric, and non-oligomeric protein species, respectively. The samples are analyzed by SDS-PAGE and the concentrations of ParF in the two fractions are determined (Barillà et al., 2005). Here, the addition of ATP or non-hydrolyzable ATPγS increased the levels of higher-order assemblies of wild-type ParF in the pellet fraction ~2-fold compared to the association levels in the absence of nucleotides (Figure 5C) which correlates with previous observations (Barillà et al., 2005, 2007; Machón et al., 2007; Dobruk-Serkowska et al., 2012; Schumacher et al., 2012). The concentrations of wild-type ParF and ParF-D111A in the pellet fractions were similar without added nucleotide. In contrast to observations with wild-type protein, both ATP and ATPγS markedly decreased the levels of ParF-D111A that entered the pellet. The ATPase activity of the mutant protein is compromised at the hydrolysis step, but not in nucleotide binding, as detailed above. As a consequence, the ineffective interaction with ATP may lock ParF-D111A into a conformation that is refractory to form higher-order structures.

ParG promotes the assembly of ParF bundles in the absence of added nucleotide, and bundling is stimulated further in the presence of ATP. ParG co-sediments partly with ParF in these reactions, but ParG alone fails to enter the pellet fraction (Barillà et al., 2005). Analogous results were observed again here: ~80% of wild-type ParF and ~50% of ParG were found in the pellet fraction, when the proteins were coincubated with ATPγS. In contrast, ParG neither stimulated the assembly of ParF-D111A nor entered into the pellet fraction when coincubated with the mutant protein (Figure 5D). These observations correlate with the impaired interaction between ParG and ParF-D111A that was evident in two-hybrid analysis (Figure 5B) and ATPase stimulation assays (Figure 4B).

## Discussion

ParF is a multifunctional ParA-type segregation protein encoded by the TP228 multidrug resistance plasmid. The protein binds and hydrolyzes ATP, assembles into higher-order species upon ATP binding, and interacts with the partner protein ParG which modulates the ATPase activity and multimerization properties of ParF (Barillà et al., 2005, 2007; Dobruk-Serkowska et al., 2012). These and other characteristics of ParF and ParG form the basis of the “Venus flytrap” mechanism of plasmid segregation: ParF forms a three-dimensional meshwork that penetrates the nucleoid interior *in vivo* where it recognizes and transports ParG-*parH* complexes and the attached plasmids to the nucleoid poles. Plasmids are deposited at the poles following the partial dissolution of the ParF network through a combination of ParG-mediated bundle disassembly and localized ATP hydrolysis within the meshwork (McLeod et al., 2017).

The “Venus flytrap” model of plasmid segregation and the action of ParA proteins more generally are contingent on the binding and hydrolysis of ATP. Residue Asp-111, which is positioned at the N-terminal end of α5 in ParF, makes crucial contacts with the ribose O3′ hydroxyl that stabilize the C3′-*endo* conformation when ParF is bound to the ATP analog, AMPPCP. Ser-108 in the preceding loop region also participates in this stabilization (Schumacher et al., 2012). Moreover, Asp-111 is part of a proline-rich region (residues 102–112) in ParF. This region in one subunit inserts into a side pocket near the nucleotide-binding pocket of the adjacent ParF subunit. These cross-contacts by the proline-rich region are proposed to stabilize the ParF-ATP sandwich dimer (Schumacher et al., 2012). The significance of Asp-111 was explored further here. First, the D111A mutation dramatically reduced plasmid segregation *in vivo*. Mutation of other residues, notably Phe-112, Gly-116, and Val-121, in α5 also negatively impacted plasmid partitioning (Figure 2A). Although the role(s) of these amino acids awaits further investigation, this triad of residues may be important in ParF subunit interactions. Second, the D111A mutation modestly affected nucleotide binding, but impaired ATP hydrolysis more conspicuously (Figure 3) which broadly agrees with a role for Asp-111 in stabilizing the C3′-*endo* pucker of the ribose ring. The sugar may undergo a switch to a less favorable C2′-*endo* conformation when alanine substitutes for aspartic acid at position 111. This physicochemical alteration in the structure of the ribose ring may influence the cleavage of the γ phosphate group in the bound ATP (Kobayashi et al., 2013). It remains to be determined whether the C2′-endo conformation is compatible with ATP binding and/or how this conformation might interfere with ATP hydrolysis by wild-type ParF. Third, ATP binding promotes ParF assembly in oligomeric bundles (Barillà et al., 2005). Nevertheless, although ParF-D111A still bound ATP effectively, formation of higher-order structures was inhibited fully *in vitro* (Figure 5C). Thus, the perturbed conformation of the nucleotide within the binding pocket of the ParF-D111A variant blocks the assembly of ParF into higher order species. Despite this inhibition, ParF-D111A dimerized in two-hybrid analysis, albeit not as effectively as the wild-type protein (Figure 5A). As a suite of amino acids are involved in dimer formation by ParF (Schumacher et al., 2012), mutation of Asp-111 alone apparently is sufficient to weaken but not abolish the two-hybrid interaction.

**Figure 6:**
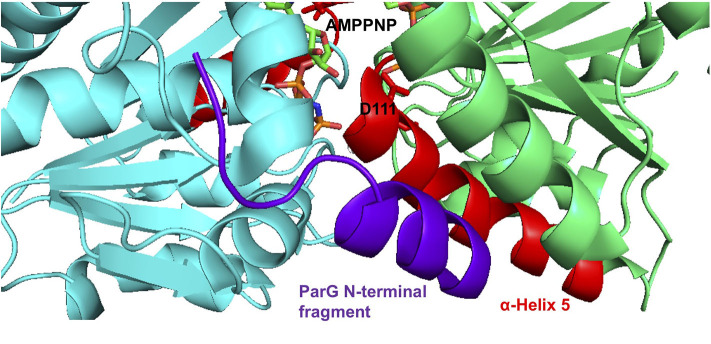
Co-crystal structure of the ParF dimer with AMPPNP and a 15 residue fragment from the N-terminal tail of ParG (Zhang and Schumacher, 2017). α-5 is highlighted in red with residue Asp-111 and AMPPNP shown as sticks. The ParG fragment is shown in purple. The images were made using PyMol (Delano, 2001).

The ParG protein plays multiple roles in plasmid segregation. The protein interacts both with the *parH* centromere during segrosome assembly and with the operator site upstream of the *parFG* genes to exert transcriptional repression (Barillà and Hayes, 2003; Carmelo et al., 2005; Zampini et al., 2009; Wu et al., 2011). DNA binding is influenced by a pair of unstructured N-terminal tails in the ParG dimer (Carmelo et al., 2005; Wu et al., 2011). The N-terminal tails also modulate both ATP hydrolysis and assembly of ParF. For the former, arginine finger-like motifs in the ParG flexible tails enhance nucleotide hydrolysis by ParF *in trans*, possibly by stabilization of the transition state through neutralization of the negative charge that develops during phosphoryl transfer (Barillà et al., 2007). However, the arginine finger-like motif in ParG did not enhance ATP hydrolysis by the ParF-D111A mutant protein (Figure 4B). The effect of the mutation on the conformation of the ribose ring in the bound nucleotide may preclude formation of the appropriate contacts by the ParG arginine finger when inserted in the ATP binding pocket. The determinants for stimulation of ParF oligomerisation by the N-terminal tails of ParG have not been defined, but are separable from the arginine finger-like motif. One or more residues within the tails are thought to play an architectural role that assists in organizing the assembly of ParF subunits into higher-order species (Barillà et al., 2007). The D111A mutation in the nucleotide binding pocket abolished the stimulation of ParF assembly in bundles by ParG (Figure 5D). This observation reveals that the block to ParF oligomerisation that is caused by the mutation is not alleviated by the stimulatory effect of the partner protein, and confirms that the conformation of the ATP binding site is critical for effective ParF assembly into higher-order structures. The profound effect that the D111A mutation has on the interaction of ParF with ParG is reflected further in the lack of detectable interaction between ParF-D111A and ParG in two-hybrid assays (Figure 5B). NMR investigation showed that residues 17–23 of the ParG N-terminal flexible tail are characterized by limited flexibility and suggested that this region might have some α -helical content (Golovanov et al., 2003). These studies also highlighted that the limited flexibility of the 17–23 region might result in entropic benefits if this fragment was to become configured rigidly when interacting with ligands, thus overall suggesting that the region might contain interaction sites. Recently, a cocrystal structure of ParF bound to a short, ~15 residue fragment of the N-terminal tail of ParG in the presence of AMPPNP was solved (Zhang and Schumacher, 2017). Although the structure has a low 3.65 Å resolution, it revealed that the ParG fragment (amino acids 8–22) folded into a short helix that was positioned next to α-helix five of ParF within a cleft at the base of the ParF dimer interface. These structural data provide evidence that ParF α-helix five is indeed part of one interface in ParF-ParG interaction (Figure 6). The absence of association observed between ParF-D111A and ParG is in agreement with the structural results and indicates that the replacement of a single residue is sufficient to disrupt the interaction interface to the point of ablating the protein-protein contact.

In conclusion, the “Venus flytrap” model of plasmid segregation necessitates the binding and hydrolysis of ATP by the ParA-type protein, ParF. The current study demonstrates that the conformation of the nucleotide binding pocket in ParF is tuned exquisitely: a single amino acid alteration that perturbs the molecular arrangement of the bound nucleotide affects not only ATP hydrolysis, but also the interaction with the partner protein ParG. The conformation of the ATP binding site in ParF profoundly impacts not only the kinetics of nucleotide hydrolysis and the capacity of an arginine finger-like motif in the N-terminal flexible tails of the ParG protein to stimulate nucleotide hydrolysis *in trans*, but also the ParF higher order dynamics that drive the segregation process. Thus, the dynamics of nucleotide binding and hydrolysis by ParA-type proteins are key to accurate genome segregation in bacteria.

## Data Availability Statement

The raw data supporting the conclusions of this article will be made available by the authors, without undue reservation, to any qualified researcher.

## Author Contributions

FH and DB conceived and designed the project with input from all authors and wrote the manuscript with input from all authors. MC, AD-S, FR-C, and CP performed the experiments and analyzed the data.

## Conflict of Interest

The authors declare that the research was conducted in the absence of any commercial or financial relationships that could be construed as a potential conflict of interest.
